# The Gut–Brain Axis in Obesity: Mechanisms, Development, and Therapeutic Perspectives

**DOI:** 10.1007/s13668-026-00732-w

**Published:** 2026-02-26

**Authors:** Laura Arellano-García, María P. Portillo, Anna Hadjihambi, J. Alfredo Martínez, Iñaki Milton-Laskibar

**Affiliations:** 1 Nutrition and Obesity Group, Department of Pharmacy and Food Sciences, Faculty of Pharmacy and Lucio Lascaray Research Centre, University of the Basque Country (UPV/EHU), Vitoria-Gasteiz, 01006 Spain; 2 CIBERobn Physiopathology of Obesity and Nutrition, Institute of Health Carlos III, Madrid, 28222 Spain; 3 BIOARABA Health Research Institute, Vitoria-Gasteiz, 01006 Spain; 4 Roger Williams Institute of Liver Studies, School of Immunology & Microbial Sciences, Faculty of Life Sciences and Medicine, Foundation for Liver Research, King’s College London, King’s College Hospital, London, UK; 5 Precision Nutrition and Cardiometabolic Health, IMDEA-Food Institute (Madrid Institute for Advanced Studies), Campus of International Excellence (CEI) UA M+CSIC, Spanish National Research Council, Madrid, Spain; 6 Centre of Medicine and Endocrinology, University of Valladolid, Valladolid, Spain

**Keywords:** Obesity, Gut-brain axis, Microbiota, Probiotics, Postbiotics

## Abstract

**Purpose of review:**

This narrative review analyses the implication of obesity-associated gut microbiota dysbiosis, as well as the associated impairments in gut-brain axis communication, in the onset and development of this chronic metabolic disease.

**Recent findings:**

Gut microbiota dysbiosis, which is a common feature in individuals with obesity, is considered among the factors leading to its development. In fact, dietary habits not only modulate the composition of gut microbiota, but also the release of microbe-derived metabolites such as SCFAs, which in turn participate in the gut-brain axis communication. Interestingly, the approaches often used for obesity management, including lifestyle modification-based interventions, pharmacotherapy or the usage of bioactives (such as phenolic compounds or probiotics) have also been shown to restore the impairments in gut-brain axis communication associated to this disease. In this regard, the recovery of gut microbiota eubiosis and improvement of the intestinal barrier function, as well as the modulation of microbial metabolite production, have been described as potential underlying mechanisms of action.

**Summary:**

The review highlights the importance of the gut-brain axis in obesity, both in the development as well as the management of this chronic metabolic disease. However, further research is warranted in order to corroborate the current findings and to better elucidate the mechanisms underlying this complex signalling network. Doing so, valuable information that will pave the way for the development of more specific and effective obesity treatments may be obtained.

## Introduction

Obesity is defined as a chronic metabolic disease characterized by an excessive or abnormal body fat accumulation that may impair health in the long term [1]. In fact, the main issue occurring in obesity is that white adipose tissue (WAT) not only increases size, but it also becomes dysfunctional, leading to chronic low-grade systemic inflammation. This is especially relevant when the excessive fat accumulation occurs in visceral depots (visceral adipose tissue, VAT), which is related to an impaired lipolysis rate, as well as to a greater production and release of pro-inflammatory mediators into the circulation. This, in turn, affects other tissues [2, 3] and organs (such as skeletal muscle or liver), leading to impairments in glycaemic homeostasis and energy substrate utilization, and thus increasing the risk of developing further obesity-associated metabolic disorders such as type 2 diabetes (T2D) and metabolic dysfunction-associated steatotic liver disease (MASLD) [3, 4].

Since obesity results from a chronic positive energy balance—shaped by genetic and non-genetic factors—the body has evolved several mechanisms to prevent excessive energy intake and thereby reduce the risk of obesity. In this regard, energy balance in the body is finely regulated by the interaction between endocrine tissues (such as the WAT) and the central nervous system (CNS) [5–7].In fact, before, during and after food consumption, different brain areas receive signals from peripheral organs, allowing for short- and long-term regulation of energy balance [8]. In such way, a correct maintenance of energy balance is guaranteed, regulating food consumption as well as the usage of the energy obtained from food digestion and nutrient metabolism. This intricate bidirectional communication network between the gut and the brain is known as the gut-brain axis and integrates neural, humoral, immune and microbial pathways. The gut-brain axis is formed by the CNS, autonomic nervous system (ANS), enteroendocrine cells (EECs) and the enteric nervous system (ENS) [9, 10]. The ENS, often referred to as the “second brain” and considered as a separated branch of the ANS, is located along the digestive tract [11]. The ENS is organised in plexuses, forming a complex circuit that coordinates local reflexes in motility and secretion and relays microbial and hormonal cues to the CNS via vagal afferents [11]. Considering the closeness of gut microbiota to the ENS, changes in microorganism composition can influence its function by direct or indirect means [11]. Indeed, in obesity, gut microbiota is impaired leading to a state often referred to as dysbiosis, which is characterized by reduced microbial diversity, enrichment of pro-inflammatory taxa, and decreased production of beneficial metabolites [12]. These alterations blunt enteroendocrine signalling, dysregulate ENS activity, and amplify systemic inflammation, ultimately impairing the gut–brain communication.

The gut microbiota is commonly defined as the complex community of microorganisms (primarily bacteria) that colonise the gastrointestinal tract. In the last years, interest in gut microbiota has increased due to its involvement in processes relevant for host’s homeostasis maintenance, such as the modulation of immune response, energy-extraction from non-digestible food components or the production of bacterial metabolites [13, 14]. In fact, the ability of gut microbiota to produce metabolites enables (in part) the aforementioned communication between the gut and the brain. For these functions to be properly carried out, gut microbiota composition must be adequate, which is commonly referred to as eubiosis. However, it has been described that individuals with obesity display dysbiosis, which is characterised by a less diverse gut microbiota, a higher production of pro-inflammatory mediators, and by an enhanced intestinal permeability (loss of the gut barrier function) [15]. In fact, due to the ability of bacterial metabolites to enter systemic circulation, and thus to participate in the crosstalk between the gut and other tissues and organs, it has been proposed that the potential impairments derived from obesity-associated gut microbiota dysbiosis may also affect the regulation of several cerebral processes [16, 17]. Likewise, the aforementioned low-grade inflammation related to obesity and dysfunctional WAT, also affects to the hypothalamic–pituitary–adrenal axis (HPA axis), which in turn may result in the impairment of energy utilization in the body [18].

In this scenario, the aim of this narrative review article is to analyse the implication of obesity-associated gut microbiota dysbiosis and gut-brain axis communication impairments in the onset and development of this chronic metabolic disease. The changes induced in gut microbiota composition by different interventions for obesity treatment, as well as the potential impact of such modulation in the gut-brain axis, are also addressed. Finally, to provide a deeper understanding of the complex relationship between the gut microbiota and the brain in obesity, the underlying mechanisms regulating this bidirectional connection are discussed.

## Gut-brain Axis and Associated Implications on the Onset and Development of Obesity

The intricate bidirectional communication of the gut–brain axis enables signals from the gastrointestinal tract to reach the CNS via neural and humoral pathways, where the continuous influx of information is integrated to generate responses that regulate energy intake and expenditure [9]. Indeed, the gut-brain axis plays a crucial role in the homeostatic control of appetite, the maintenance of an adequate energy balance and the control of energy expenditure [9]. More specifically, even before their absorption, nutrients generate signals (through mechanisms that will be described in more detail below) along the gastrointestinal tract, providing information about the energy density and nutritional composition of a given meal. All this information is then processed in specific brain-areas, resulting in responses involving energy intake and expenditure regulation, and thus enabling an adequate energy homeostasis during periods of food intake and fasting [9].

Vagal afferents do not directly contact luminal content but instead sense nutrients and microbial metabolites indirectly via absorbed compounds or through signals from epithelial cells, including EECs [19]. In fact, and despite representing only 1% o the epithelial cells present in the gastrointestinal tract, EECs are responsible for making the gut the largest endocrine organ in humans [20]. EECs are distributed throughout the gastrointestinal epithelium and present a microvilli-covered apical membrane, which directly contacts the luminal content of gastrointestinal tract [21]. After EECs are stimulated by nutrients or metabolites, a range of hormones are secreted. Of note, the secretome of EECs depends on their location, as stomach EECs secrete orexigenic hormones such as ghrelin or gastrin, while intestinal EECs release anorexigenic hormones such as glucagon-like peptide-1 (GLP-1), cholecystokinin (CCK) and peptide YY (PYY) [20, 21]. The released products activate a wide range of receptors located in neighbouring EECs, immune cells, target organs or afferent nerves (vagal or ENS nerves), or are directly released into systemic circulation [20].

**Fig. 1:**
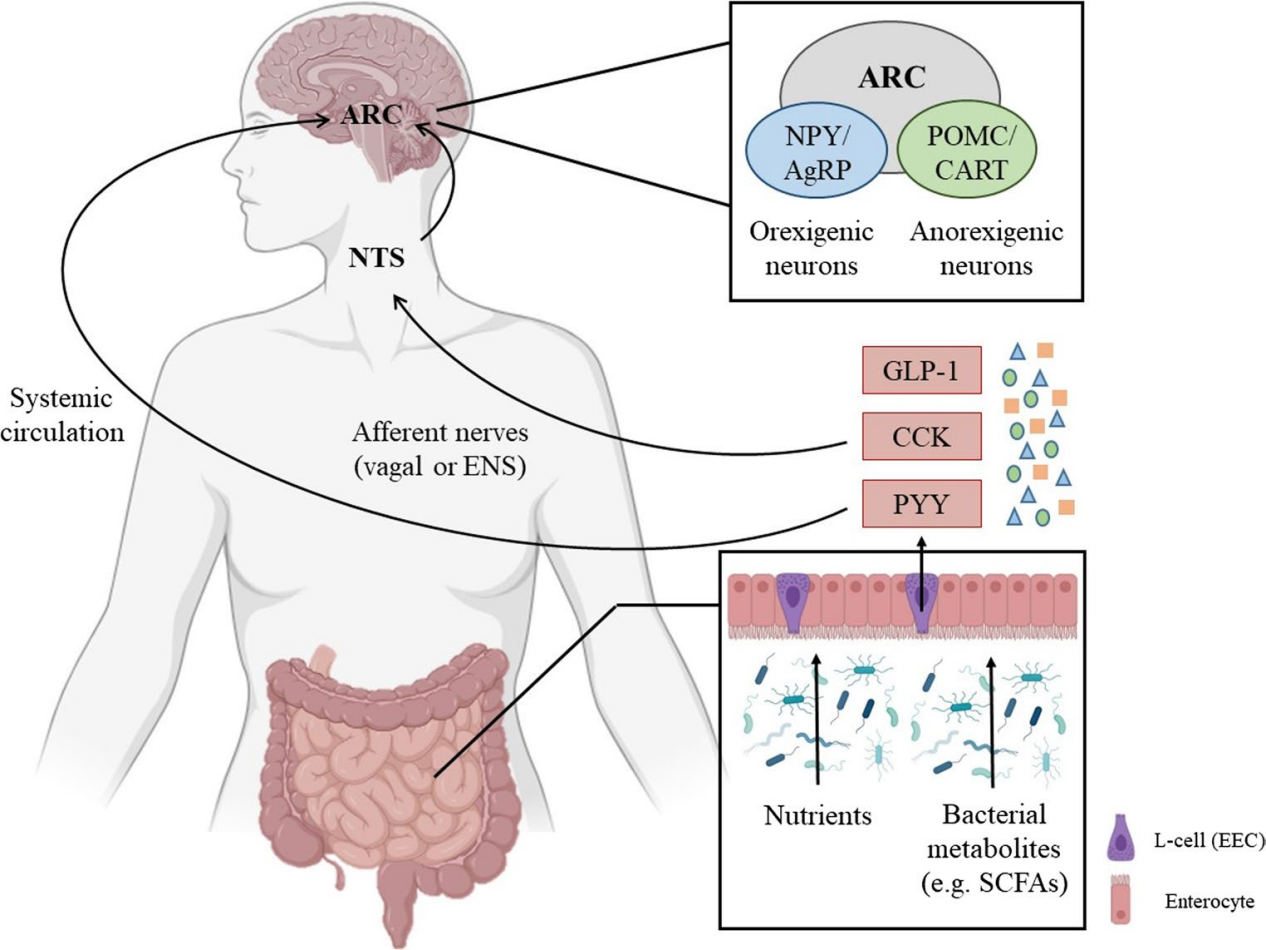
Schematic representation of the gut-brain axis humoral signalling network. AgRP: agouti-related protein, ARC: arcuate nucleus, CART: cocaine- and amphetamine-regulated transcript, CCK: cholecystokinin, ENS: enteric nervous system, GLP-1: glucagon-like peptide 1, NPY: neuropeptide Y, NTS: nucleus of the solitary tractus, POMC: pro-opiomelanocortin, PYY: peptide YY, SCFAs: short-chain fatty acids

These nutrient-derived signals are conveyed to the brainstem, more specifically to the nucleus of the solitary tract (NTS), which integrates stimuli and innervates a range of brain regions, such as the arcuate nucleus (ARC), located in the hypothalamus [22, 23]. The ARC has been recognised as a central hub for regulating feeding behaviour and energy homeostasis [21]. This hypothalamic region responds to appetite signals by releasing neurotransmitters from two neuronal populations: orexigenic neurons, which stimulate appetite by secreting neuropeptide Y (NPY) and agouti-related protein (AgRP), and anorexigenic neurons, which trigger satiety by secreting pro-opiomelanocortin (POMC) and cocaine- and amphetamine-regulated transcript (CART) [24]. Of note, as mentioned before, EEC-derived hormones may be directly secreted into circulation (humoral signalling), thereby reaching the ARC and contributing to energy homeostasis regulation (Fig. 1) [21].

In the last decades, gut microbiota has been recognized as an additional player in gut-brain axis. A bidirectional interaction has also been established between the CNS and gut microbiota, which produces a range of metabolites such as short-chain fatty acids (SCFAs), lipopolysaccharide (LPS), catecholamines, 5-HT or γ-aminobutyric acid (GABA), that influence overall energy intake by regulating appetite and satiety through similar pathways as nutrients (both humoral and neural) [25, 26]. Indeed, while EECs activation on the proximal intestine (duodenum) is triggered primarily by nutrients, distal EECs (located on jejunum, ileum, colon and rectum) are almost exclusively activated by bacteria-derived metabolites [25, 27]. For instance, SCFAs derived from the fermentation of indigestible non-absorbable fibre (mainly acetate, butyrate and propionate), have been extensively studied by their physiological effects [28–30]. Additionally to their known local effects in the colon (improvement of intestinal barrier integrity, enhancement of mucus production and anti-inflammatory effect, among others), SCFAs seem to play a crucial role in gut-brain crosstalk [31]. In this regard, SCFAs are known to bind to G-protein coupled receptors (GPCRs), such as fatty-acid receptor FFAR2/3 (GPCR43/41), which are expressed in EECs, triggering the release of hormones including GLP-1 or PYY [32].These hormones influence eating behaviour, including hunger, satiety and mechanisms regulating appetite, as explained earlier [33].

Apart from SCFAs, gut bacteria also produces bioactive lipids related to endocannabinoids. For example, *Akkermansia muciniphila* has been shown to increase the levels of *N*-oleoylethanolamine and 2-oleoylglycerol, which enhance the secretion of GLP-1 [34]. Finally, other mediators are also involved in the crosstalk between the gut microbiota and the CNS to control food intake. Thus, some intestinal bacteria may produce protein sequences identical to appetite-regulating peptides such as leptin, PYY, ghrelin or α-melanocyte-stimulating hormone (α-MSH) [35]. Studies have described that caseinolytic peptidase B protein homolog (ClpB) mimics the anorexogenic effect of α-melanocyte-stimulating hormone α-MSH, and enhances PYY and GLP-1 secretion, directly activating anorexigenic neurons and promoting satiety [35]. Of note, apart from the effects of gut microbiota-derived metabolites in regulating food behaviour, the CNS may also modulate bacterial composition and function either directly, through the secretion of signalling molecules, or indirectly, by altering the gut environment [36].

## The “Obese Microbiota” and the Gut-Brain Axis

Although obesity is a multifactorial disease, diet plays a crucial role in its pathophysiology. Indeed, variations in diet are thought to explain up to 57% of the structural changes (taxonomic composition, diversity, community balance and functional potential) in total gut microbiota [22]. Thus, dietary habits modulate the composition of gut microbiota, which partly determines the effects of ingested nutrients through its processing and the release of microbe-derived metabolites [37]. Observational studies have reported that individuals with overweight or obesity display different microbiota signatures [37]. Interestingly, variations have also been observed on individuals suffering from obesity-related co-morbidities (such as insulin resistance, hypertension or cardiovascular disease) [37]. Moreover, studies performed with germ-free mice receiving the gut microbiota of either mice or humans with obesity, developed the host phenotype, highlighting the link between gut microbiota composition and the development of associated metabolic impairments [38].

Generally, gut microbiota signature in individuals showing obesity is characterized by a lower microbial diversity, as well as by an altered relative abundance of specific bacterial taxa such as *Akkermansia* spp., *Christensenella* spp., *Bacteroides* spp., *Prevotella* spp. or *Blautia* spp [37]. These changes are usually accompanied by a shift in metabolic pathways and microbiota-derived products, such as the levels or the type of released SCFAs or an impaired production of ethanol and LPS [37]. It is important to note that obesity is not characterized by a single, specific gut microbiota signature. Instead, it is associated with a constellation of compositional and functional alterations, collectively referred to in this manuscript as dysbiosis. As gut microbiota composition and its secretome are both implicated in gut-brain communication pathways, their alteration also leads to changes in the functionality of such axis, which in turn has been related to obesity development [37]. Indeed, individuals with obesity exhibit impairments in both the homeostatic and hedonic pathways regulating food-related behaviour, which may contribute to dysfunctional eating patterns and, ultimately, overfeeding [39].

Considering the role of EECs in the regulation of food intake, the link between gut microbiota dysbiosis and EEC function has generated renewed interest. A study using high-fat fed mice showed that such feeding pattern drove changes in gut microbiota composition, which were accompanied by modifications in ECC genes related to GLP-1 expression [40]. These results highlight the importance of gut microbiota eubiosis in EEC function, with dysbiosis being linked to an impaired hormone production and release in these cells. Indeed, individuals with obesity also display dysregulated EEC-hormone secretion, possibly contributing to the alteration of energy balance [27, 37]. More specifically, GLP-1, CCK and PYY levels have been shown to be lowered in individuals with obesity [41–43], something that has been related to an increased appetite and food intake [44].

This impaired hormone secretion in ECC cells seems to be mediated through different mechanisms. First, a reduction in the number of EECs has been observed in individuals with obesity compared to lean controls, which most likely may affect hormone production and secretion [43]. Additionally, it has also been reported that individuals with obesity overexpress a negative regulator of GPCR signalling. This alteration may contribute to a reduced sensing of microbe-derived metabolites and nutrients, and thus to a decreased GLP-1 and PYY secretion after meal intake, ultimately affecting glucose metabolism and food intake [45]. This is supported by observations reporting that individuals with obesity derived from the consumption of a hypercaloric diet, display an altered nutrient sensing [37]. Interestingly, EEC deficiency leads to the alteration of gut microbiota composition, highlighting the bidirectional communication between intestinal microorganisms and gut [46].

## Effects of Body-Weight Management Interventions on the Gut-Brain Axis Signalling

Given the established relationship between gut microbiota and obesity, and the role of the gut–brain axis in regulating energy balance and food intake, an additional area of interest concerns the impact of body-weight management interventions on this communication network. Such interventions may not only modulate gut microbiota composition but also contribute to restoring the gut–brain crosstalk, which is often disrupted in obesity.

### Lifestyle Modification Based Interventions

Considering that obesity is the result of an imbalance between energy intake and expenditure maintained in time, one of the most used therapeutic approaches relies on lifestyle modification interventions. Therefore, dietary interventions involving restriction of total caloric intake and/or restriction of meal consumption time, along with an enhanced practice of physical activity, has been conventionally the “go-to” strategy to tackle obesity. In this context, while a ‘one-size-fits-all’ intervention may vary in its effectiveness for obesity treatment, research has increasingly focused on its impact on gut microbiota composition and, consequently, on the gut–brain axis.

Caloric-restriction (CR) is the most commonly used strategy when referring to dietary interventions for obesity management. This approach is commonly based on the reduction of 20–40% of total daily energy intake, without compromising a suitable nutrient intake. Besides its obvious application for obesity management, CR has long been considered as a potential life extending intervention based on results obtained from various experimental models [47, 48]. Regarding the impact of CR on gut microbiota, several studies have described an enrichment of *Bacteroidetes* and a decrease in *Firmicutes* (and thus a reduction in the *Firmicutes*/*Bacteroidetes* ratio) relative abundances in preclinical studies and clinical trials [49, 50]. Likewise, the relative abundance of *Akkermansia muciniphila* is another indicator that is commonly upregulated following CR-based interventions [51, 52]. These Gram-negative anaerobic bacteria have the ability to degrade mucin, providing energy for a correct maintenance of intestinal barrier function. Consequently, an enhanced relative abundance of *Akkermansia muciniphila* has been related to better metabolic outcomes in individuals with overweight or obesity, undergoing CR [53]. Other authors have also reported variations in the relative abundances of bacteria such as *Lactobacillus*, *Bifidobacterium*, *Roseburia* or *Coprococcus* in humans following CR interventions, despite no consensus regarding these effects has been reached so far (mainly due to baseline variations in gut microbiota composition) [54]. Additionally, and according to the available data, CR not only affects gut microbiota composition, but also its function. In this regard, it has been described that CR promotes functions favouring the production of propionate, while limiting the production of acetate and butyrate [54].

All these CR-derived changes seem to have an overall positive impact on gut microbiota composition (enrichment of beneficial bacteria), gut barrier function (reducing intestinal permeability) and intestinal inflammatory status (modulating microbial metabolite production). This, in turn, has a direct effect on the gut-brain axis communication, which is impaired in obesity. Firstly, the abundance of LPS producing bacteria, and thus of LPS, are reduced under CR. Considering that LPS-mediated endotoxemia negatively affects brain inflammation, leading to the activation of the nuclear factor kappa-light-chain-enhancer of activated B cells (NFκB) pathway and the production of pro-inflammatory cytokines (mainly tumour necrosis factor α, TNFα, and interleukin 1β, IL-1β), this CR-mediated effect may help restore the communication between the gut and the brain [25]. Noteworthily, the aforementioned improvement in gut barrier function occurring in CR may also be of help in reducing the potential flux of LPS from the gut into the circulation. In addition, the modulation of SCFA production occurring in CR has also an impact in the gut-brain axis. In this regard, SCFA have the ability to reduce intestinal inflammatory status (counteracting the effects of other pro-inflammatory mediators such as LPS) as well as to enhance gut barrier function, serving as energetic substrates for colonic epithelial cells [25]. Likewise, CR-mediated SCFA modulation also affects satiety through the production of anorexigenic peptides in the gut (such as PYY and GLP-1) as well as entering systemic circulation and reaching the brain [55].

Another type of dietary intervention for obesity management that has gained much attention is fast-mimicking diets. These diets are characterised by the timed consumption of energy-providing macronutrients within a specific eating window. A key advantage of these diets over conventional hypocaloric approaches is that they do not require calorie counting during the restricted eating window (there is no caloric restriction), thereby promoting greater adherence to the intervention. The most common fast-mimicking diets are intermittent fasting (alternating eating days with fasting days) and time-restricted eating (based on meal consumption time restriction within 24 h). Besides their usefulness for obesity management, different studies have also analysed their impact on gut microbiota. For instance, results obtained in preclinical studies indicate that intermittent fasting is related to greater relative abundances of *Lactobacillus* and *Bifidobacterium* [54]. In addition, studies conducted in humans undergoing intermittent fasting revealed that this approach affects bacterial taxa involved in SCFA production [56]. Similar results have been reported from subjects undergoing time-restricted eating regimes, such as the Ramadan, where higher abundances of butyric producing bacteria (including *Ruminococcaceae* and *Lachnospiraceae* families) and enhanced circulating butyric acid levels have been described [57, 58]. Noteworthily, clinical trials involving individuals who have obesity, undergoing time-restricted eating interventions, have also related this dietary pattern to higher abundances of SCFA producing bacteria [59]. Current evidence suggests that fast-mimicking diets may help restore a healthier gut microbiota composition in individuals with obesity. Moreover, as these microbiota shifts are related to an enhanced production of SCFA (mainly butyric), a role in regulating the gut–brain axis via modulation of EEC hormone secretion (as described for CR) cannot be excluded [33].

Besides dietary intervention, the practice of physical activity is the other key component of lifestyle modification based interventions for the management of obesity. In this regard, general recommendations indicate that the practice of 150–300 min/week of moderate-intensity aerobic physical activity helps reduce body-weight, while improving metabolic outcomes that are usually impaired in individuals with obesity (such as glycaemia or arterial blood pressure [60]. Recent evidence suggests that the benefits of physical activity in obesity may be mediated, at least in part, through its modulatory effects on gut microbiota composition. Regular practice of moderate aerobic physical activity (as prescribed for obesity management) promotes a more diverse gut microbiota composition, enhancing the relative abundance of butyrate producing bacteria and *Akkermansia muciniphila* [61]. Interestingly, these benefits seem to be independent of diet and disappear once the practice of physical activity stops [62]. Likewise, physical activity also modulates the production of bacterial metabolites and gut peptides that ultimately are involved in appetite/satiety regulation. It has been suggested that acute exercise practice may help control satiety in individuals with overweight and obesity by increasing circulating GLP-1 and PYY levels, as well as by suppressing acetylated ghrelin [63]. Besides the aforementioned physical activity-mediated effects on gut microbiota and the gut-brain axis, it must be noted that skeletal muscle can act as an endocrine tissue. In fact, skeletal muscle can produce and release a series of molecules called myokines (including irisin and myonectin, among others), as well as PYY, into the bloodstream and therefore contribute to appetite regulation. However, there are certain variables associated to physical activity, such as the exercise type (aerobic or anaerobic), intensity and duration that can influence the impact of physical activity on gut microbiota (and thus should be considered) [64]. In fact, high-intensity exercise seems to negatively affect gut microbiota composition and intestinal integrity, leading to impairments in bacterial metabolite production (towards a more pro-inflammatory species secretion) and increasing intestinal permeability [61].

**Fig. 2:**
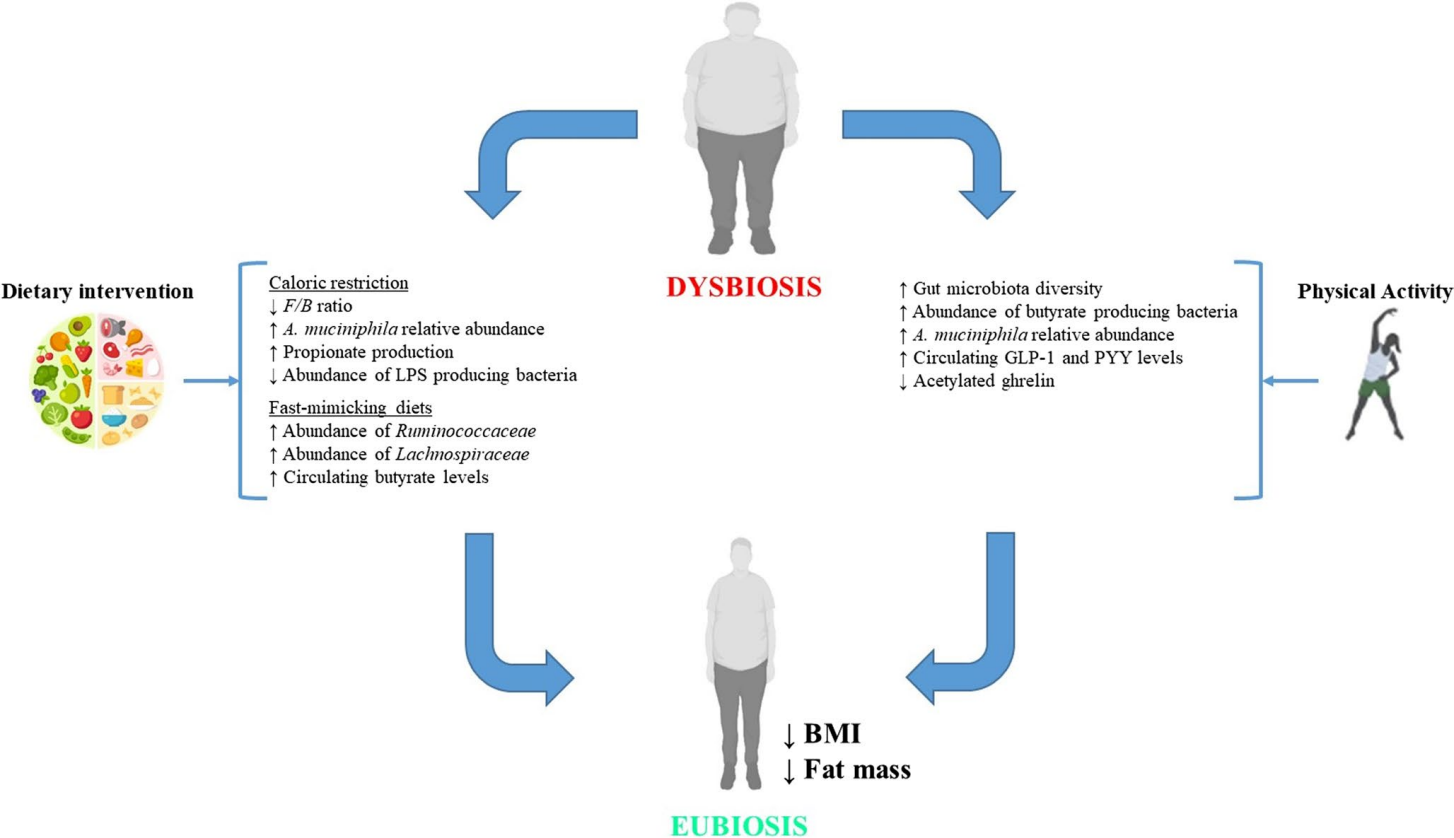
Schematic representation of the effects of lifestyle-modification based intervention for obesity management on gut microbiota and the gut-brain axis. *A. muciniphila*: *Akkermansia muciniphila*, BMI: body-mass index, *F/B*: *Firmicutes*/*Bacteroidetes*, GLP-1: glucagon-like peptide, LPS: l ipopolysaccharide and PYY: peptide YY

To sum up, the current evidence from clinical studies indicates that both components of lifestyle modification-based interventions for weight-loss can have a positive impact on gut microbiota, and thus may help restore the communication along the gut-brain axis (Fig. 2). Besides the apparent benefits of this kind of approach regarding body-weight management and the crosstalk between the gut and the brain, variability on baseline subject characteristics (including age, sex or weight), the nature of the selected dietary intervention (with or without CR) or the proposed physical activity program (type of exercise, intensity and duration) are factors that may affect the outcome of the intervention, and thus must be considered.

### Pharmacological Treatment of Obesity

Pharmacological therapy represents another strategy for obesity management, although it is generally not considered a first-line option. Current guidelines recommend its use in individuals with a body mass index (BMI) > 30 kg/m^2^, or having a BMI > 27 kg/m^2^ with weight-related comorbidities and following failure achieving clinically significant weight loss (≥ 5% baseline weight) after 6 months of lifestyle interventions [65]. To be approved for obesity treatment, drugs must induce a ≥ 5% reduction in body weight within one year and demonstrate efficacy in at least 35% of treated individuals compared with placebo.

This review article will only consider approved anti-obesity drugs that are used for the treatment of non-syndromic obesity since syndromic obesity only represents a small fraction of all obesity cases worldwide (Table 1).

**Table 1:** Drugs approved for non-syndromic obesity pharmacotherapy and their effects on gut microbiota

Reference	Drug	Anti-obesity mechanism of action	Effect on gut microbiota
[66, 67]	Orlistat	Inhibition of gastric and pancreatic lipases • ↓ Dietary TG hydrolysis	↑ Microbial diversity ↓ *F/B* ratio ↑ Relative abundance of *Lactobacillus* genus ↑ HYA, KetoA and KetoC production
[68]	Liraglutide	GLP1-R agonist • Appetite regulation	↑ Gut microbiota diversity and richness ↑ Relative abundance of *Bacteroidetes*, *Proteobacteria* and *Bacilli*
[69]	Semaglutide	GLP1-R agonist • Appetite regulation	No available data from clinical studies
[70]	Naltrexone/Bupropion	Antagonist of opioid receptors (Naltrexone) Inhibition of norepinephrine and dopamine reuptake (Bupropion) • ↑ Production of POMC peptide	↑ Gut barrier function (Naltrexone alone) ↓ Relative abundance of *Roseburia* genus
[65]	Phentermine/Topiramate	Stimulation of epinephrine and norepinephrine release (Phentermine) Inhibition of carbonic anhydrase (Topiramate) • Mechanism unknown (↓ appetite)	No available data from clinical studies

*F/B*: *Firmicutes*/*Bacteroidetes*, HYAC: 10-Hydroxy-*cis*−12-octadecenoic acid, GLP1-R: glucagon-like peptide 1 receptor, POMC: pro-opiomelanocortin, TG: triglyceride, ↑: significant increase, ↓: significant decrease

One of the most frequently prescribed drugs for obesity pharmacotherapy is orlistat, a reversible inhibitor of gastric and pancreatic lipases that blocks dietary triglyceride (TG) hydrolysis, thus reducing fat absorption by up to 30% [71]. Long-term clinical trials have demonstrated that orlistat treatment effectively reduces body-weight in individuals with obesity undergoing a lifestyle modification intervention (physical activity and CR). Moreover, the combination of orlistat with either, antidiabetic drugs (such as metformin) or insulin, seems to promote a greater body-weight loss (compared to orlistat alone) in individuals with overweight or obesity [72, 73]. Besides the benefits on body-weight management and in the improvement of different metabolic outcomes, orlistat administration has also been associated with an enhanced microbial diversity, a decreased *Firmicutes*/*Bacteroidetes* ratio and an increased abundance of *Lactobacillus* genus in individuals with visceral obesity and receiving the drug for 8 weeks [66]. In addition, increased linoleic acid metabolites such as 10-hydroxy-*cis*−12-octadecenoic acid (HYA), ketoA and ketoC were also found in this same cohort after orlistat treatment. Indeed, this regard, it is known that HYA can interact with G-protein coupled receptor 40 (GRP40), thus suppressing the production of pro-inflammatory cytokines and restoring the intestinal barrier function [67]. Likewise, it has been reported that ketoA triggers energy expenditure (through the activation of capsaicin receptor), whereas ketoC reduces LPS-mediated TNFα and interleukin 6 (IL-6) production [66]. All these orlistat-mediated effects may positively impact the gut-brain axis in obese subjects under pharmacotherapy, and thus help to regulate body-weight. By contrast, other clinical trials involving patients with obesity, showing dyslipidaemia, and undergoing a 12-week treatment with orlistat and ezetimibe (a selective cholesterol absorption inhibitor) revealed no significant changes in gut microbiota composition nor in SCFA levels [74]. Similar results were described in a study carried out in healthy individuals following a 1 week orlistat treatment. Also in this case, no relevant changes in gut microbiota composition or stool SCFA levels were found at the end of the study compared to baseline [75]. Thus, despite the established benefits of orlistat for obesity treatment, its effects on gut microbiota are not as clear. In this regard, variables such as the characteristics of the individuals at baseline, as well as the lipid content and type of the diet during orlistat treatment may influence the impact of the treatment on gut microbiota.

Liraglutide and semaglutide, both GLP-1 receptor (GLP-1R) agonists, are widely used in obesity pharmacotherapy. Originally developed as antidiabetic agents, these drugs have more recently demonstrated significant efficacy in obesity management by promoting weight loss through appetite suppression, enhanced pancreatic insulin secretion, delayed gastric emptying, and reduced intestinal motility [69]. According to data obtained in clinical trials ranging from 56 to 160 weeks, and including lifestyle modifications (physical activity advice and CR), such as the Satiety and Clinical Adiposity Liraglutide Evidence program, liraglutide administration resulted in a body-weight loss of 8 and 6,1% of the baseline weight (after 56 and 160 weeks of treatment, respectively) in individuals with obesity [76, 77]. Likewise, data from the Semaglutide Treatment Effect in People with Obesity trials demonstrate that semaglutide administration for 68 weeks (in combination with dietary advice and increased practice of physical activity) is an effective approach for body-weight reduction in individuals with obesity, and that this effect is also maintained in individuals with obesity-associated co-morbidities (such as diabetes mellitus) [78, 79].

The available evidence regarding the impact of GLP1R agonists on gut microbiota is scarcer and mainly derived from preclinical studies. In this regard, preclinical studies have demonstrated that semaglutide effectively improved high-fat diet induced dysbiosis in obese mice, triggering the relative abundance of beneficial bacteria such as *Akkermansia muciniphilla* [80]. As for liraglutide, preclinical studies described that its administration modulates gut microbiota composition towards a lean-related profile (enriched in bacteria from the genera *Lactobacillus*, *Blautia* and *Turicibacter*) in diabetic mice with obesity and spontaneous type 2 diabetic rats [81, 82]. Indeed, decreased *Firmicutes*/*Bacteroidetes* ratio, along with an increased relative abundance of *Akkermansia muciniphila* have also been described in obese murine models receiving this drug [68].

Concerning evidence from clinical trials, liraglutide administration seems to enhance gut microbiota diversity and richness, while promoting the relative abundance of specific bacteria (including *Bacteroidetes*, *Proteobacteria* and *Bacilli*) in individuals with obesity, featuring diabetes and steatotic liver [83]. By contrast, there is currently no published clinical data regarding the effect of semaglutide administration on gut microbiota. Due to the insufficient evidence regarding the impact of GLP1R agonists on gut microbiota, the impact that these anti-obesity drugs may have on the gut-brain axis remains unknown.

Another approach for obesity pharmacotherapy is based on the concomitant administration of naltrexone (antagonist of opioid receptors) and bupropion (inhibitor of norepinephrine and dopamine reuptake), which results in an increased production of POMC peptide, and thus, in a reduction in food intake [65]. Additionally, administration of naltrexone/bupropion also affects reward pathways, and thus improves self-control through the activation of specific brain areas [84]. In the case of naltrexone, its usage is related to a reduction in the subjective liking of specific foods (usually the most palatable ones), affecting the overall food intake and thus reducing its body-weight. The mechanisms underlying bupropion’s effects on body-weight reduction in humans remain incompletely understood. However, preclinical studies in obese rodent models have reported reduced food intake and increased energy expenditure, the latter mediated by enhanced thermogenesis [85]. Regarding the anti-obesity effects of the naltrexone/bupropion combination, evidence from randomized clinical trials, including the Contrave Obesity Trials program, shows that administration of varying doses alongside lifestyle modification over 56 weeks produced a dose-dependent body-weight reduction of approximately 5–6.4% from baseline in adult men and women with overweight or obesity [86, 87]. Concerning the impact of naltrexone/bupropion administration on gut microbiota composition, the available evidence is scarce and mainly derived from preclinical studies. It has been reported that the administration of naltrexone/bupropion for 42 days increased *Bacteroidetes* phylum member abundance (as well as the abundance of specific bacteria) in female rats with obesity fed with a high-fat diet. Interestingly, the changes induced by the combined administration of naltrexone/bupropion on gut microbiota were not observed when administering the drugs separately [88]. In humans, the consumption of naltrexone alone has been related to improvements in gut barrier function. Likewise, it has been reported that the relative abundance of bacteria from the *Roseburia* genera (which are butyrate producers) is lower in individuals using opioid agonists in comparison to those using naltrexone [70]. However, since these studies were not conducted in subjects with obesity, and in some cases the usage of drugs was self-reported, the aforementioned observations are yet to be corroborated in future clinical trials (preferably individuals with obesity). To the best of our knowledge, no studies have been carried out analysing the effects of bupropion or naltrexone/bupropion administration on human gut microbiota composition.

The combined administration of phentermine and topiramate is another approach that has been approved for long-term obesity pharmacotherapy. Phentermine is a sympathomimetic anorectic amine that stimulates epinephrine and norepinephrine release, while topiramate is an inhibitor of carbonic anhydrase, a GABA agonist and glutamate antagonist. While the exact mechanisms of action underlying the anti-obesity effects of phentermine/topiramate administration are not fully known, their usage has shown to effectively suppress appetite [65]. Moreover, and according to data obtained in preclinical studies, sole administration of topiramate also affects energy balance, triggering thermogenesis in brown adipose tissue [89]. However, to date such effects are yet to be confirmed in humans. As for the anti-obesity effect of phentermine/topiramate, the available data obtained from randomised clinical trials using different doses and treatment periods (ranging from 52 to 56 weeks) demonstrate that the administration of phentermine/topiramate (along with standardized counselling for diet and lifestyle modification) induces body-weight reductions up to 10,5% of baseline weight in adults with obesity [90, 91]. With regard to gut microbiota, a study conducted in diet-induced obese mice revealed that sole administration of phentermine reverted the diet-induced increase in the *Firmicutes*/*Bacteroidetes* ratio, while restoring the abundance of *Oscillospiraceae*, *Ruminococcaceae* and *Prevotellaceae*, reaching values similar to those observed in the control animals. In other preclinical studies, although no changes in α-diversity were observed, an increased abundance of Lactobacillus johnsonii was detected in the fecal samples of mice treated with this drug for 5 weeks. However, it must be noted that this study was not conducted in animal models of obesity, and thus the effects of topiramate in obesity-associated dysbiosis is still unknown [92]. As for the effects of the combined administration of phentermine/topiramate on gut microbiota, to the best of our knowledge no study (preclinical or clinical) has been published so far.

Taken together, the available evidence indicates that the drugs currently prescribed for obesity pharmacotherapy have a positive impact on gut microbiota dysbiosis commonly present in this chronic metabolic disease. Likewise, the changes in gut microbiota composition induced by the aforementioned anti-obesity drugs usually lead to the modulation of bacterial metabolite production and release, which in turn may positively affect communication along the gut-brain axis. Nevertheless, the different nature of the analysed anti-obesity drugs, as well as other factors including patient traits (degree of obesity, sex or age) or treatment characteristics (dosage, treatment length or additional dietary/lifestyle advice) may also influence not only the outcome of the treatment in terms of body-weight reduction, but also regarding its impact on gut microbiota and the gut-brain axis.

In addition, other pharmacological treatments, non-related to obesity, that can often be used by individuals with overweight also induce modifications in gut microbiota composition and function. For instance, it is well known that antibiotics-based treatments negatively affect the composition and function of gut microbiota, which in turn may result on impairments of the immune system [93]. Likewise, the consumption of non-steroidal anti-inflammatory drugs (NSAIDs) like ibuprofen or sodic naproxen has also been associated with impairments in gut microbiota composition (mainly affecting the relative abundance of Gram-negative bacteria) [94].

Finally, it must also be considered that besides being affected by the consumption of drugs, gut microbiota itself is also involved in drug response and metabolism [95].

### Bariatric Surgery

Besides the aforementioned interventions, surgical treatment of obesity (namely bariatric surgery) is another therapeutic approach that promotes significant body-weight loss in individuals with obesity. However, bariatric surgery is not usually the first therapeutic option for obesity management since patients need to meet certain criteria (BMI score ≥ 40 kg/m^2^ or ≥ 35 kg/m^2^ with concomitant obesity related co-morbidities) to be eligible for this kind of intervention [96]. Moreover, besides the effectiveness of bariatric surgery to significantly reduce body-weight (up to 25 to 35% in 1–2 years), this intervention has also been associated with improvements in obesity-associated co-morbidities, such as type 2 diabetes or cardiovascular risk factors [97] and quality of life [98]. Indeed, evidence indicates that bariatric surgery derived improvements in glycaemic control are superior to those exerted by lifestyle-modification based interventions or obesity pharmacotherapy [99].

There are different types of interventions within bariatric surgery that have been maintained over time, demonstrating their usefulness and safety for obesity treatment. Among them, sleeve gastrectomy, gastric banding and Roux-en-Y gastric bypass are the most commonly used ones. While these surgical interventions were originally designed to physically limit food intake (reducing the size of the stomach) and/or impairing nutrient absorption from ingested food, available evidence indicates that the contribution of these mechanisms of action to body-weight reduction is lower than one could expect [100]. In fact, it has been demonstrated that bariatric surgery also leads to neuroendocrine modulation involving gut hormone production [97]. For example, in purely restrictive bariatric surgery interventions, a reduction in the levels of orexigenic hormones and increased levels of incretins has been reported [96].

The current knowledge regarding the effects of bariatric surgery on gut microbiota is limited since available studies addressing this topic are still scarce. Similarly, the available results do not fully clarify whether bariatric surgery–induced alterations in gut microbiota are driven by changes in food intake and digestion, by the surgical procedure itself, or by the associated metabolic improvements, such as reductions in systemic inflammation [96]. Additionally, it must be noted that the type of conducted bariatric surgery intervention may have a different impact/effect on gut microbiota [101]. Overall, the available evidence indicates that bariatric surgery results in an increased gut microbiota richness and diversity, an observation that has been corroborated in studies using different techniques to analyse these parameters. More specifically, significant changes in the relative abundances of bacteria from the genera *Ruminococcaceae*, *Faecalibaceterium* and *Eubacterium* have been described, which in turn are related to an ameliorated metabolic status. However, it must be noted that the obesity-associated gut microbiota dysbiosis, that is usually present in patients undergoing bariatric surgery, is not fully reversed by these surgical interventions [101]. Noteworthily, the gut microbiota composition modulation observed in individuals undergoing bariatric surgery seems to be maintained over long periods of time (up to 9 years) [102]. Overall, the impact of bariatric surgery on gut microbiota is considered positive, although the exact mechanisms of action that may explain these changes remains unclear [103].

Besides affecting gut microbiota composition, bariatric surgery has also influence on the gut-brain axis communication network. As indicated previously, the neuro hormonal changes occurring following bariatric surgery interventions seem to explain (at least in part) the post-operative weight-loss. Firstly, it has been described that bariatric surgery induces changes in the CNS, which in turn are related to energy balance and food intake regulation. Thus, bariatric surgery seems to affect hypothalamic NPY expression, despite the available evidence is unclear. Likewise, it has been proposed the implication of the melanocortin-4 receptor (MC4R) signalling pathway in the body-weight loss following bariatric surgery. In this regard, it seems that the greater gut-hormone expression occurring after bariatric surgery (mainly increased PYY and GLP-1) may trigger MC4R production in the CNS, and thus reduce food intake.

Additionally, the available scientific evidence also suggests that bariatric surgery also induces changes in the autonomous nervous system, affecting food intake and energy expenditure. Despite a considerable part of the literature on this issue derives from preclinical studies, and sometimes the obtained results are contradictory or unclear, it seems that bariatric surgery may trigger energy expenditure increasing brown adipose tissue (BAT) sympathetic activity, and thus enhancing thermogenesis [104]. Other authors also reported that the potential damages that may occur in the vagal nerve during bariatric surgery result in changes on its hormonal sensitivity, leading to a reduction in food intake [105].

It has been described that bariatric surgery significantly affects the circulating levels of a variety of gut hormones that participate in the regulation of the eating-behaviour acting along both humoral and neural pathways of the gut-brain axis (need a ref). Enhanced CCK levels have been described following certain bariatric surgery interventions, which may derive from a potential post-operative proliferation of CCK producing cells or an increased release of factors promoting CCK release [97, 106]. Similarly, the available evidence indicates that PYY levels are also increased in individuals undergoing bariatric surgery, and that this effect is maintained regardless of the type of surgery [97]. Moreover, several studies have found that post-prandial circulating levels of GLP-1 are enhanced in individuals following bariatric surgery, an effect that seems to be mediated by an increased expansion of GLP-1 expressing cells in the intestine [107]. Interestingly, several studies have reported a correlation between postoperative body-weight loss and circulating GLP-1 levels, with greater weight reduction observed in individuals exhibiting more pronounced increases in this hormone [108]. Oxyntomodulin is another gut-hormone that is known to regulate food intake (reducing appetite and food intake) via GLP1R. According to the available data, increased post-prandial oxyntomodulin levels have been described in subjects undergoing Roux-en-Y gastric bypass, results that have not been observed in other bariatric surgery interventions [109]. By contrast, the effects of bariatric surgery in the levels of other hormones are more controversial. For instance, there is no consensus regarding the impact of bariatric surgery in ghrelin levels (a hormone that triggers hunger) since various studies have reported opposed results that also seem to be influenced by the type of surgical intervention [97]. Likewise, some authors described increased post-prandial levels of pro-uroguanylin (a pro hormone that activates POMC, suppressing appetite) in bariatric surgery patients, with this increase not correlating with reduced hunger perception [110]. Finally, the greater gut-hormone expression occurring after bariatric surgery (mainly increased PYY and GLP-1) may trigger the melanocortin-4 receptor signalling pathway in the CNS, and thus reduce food intake, contributing to the reduction of body weight post-surgery [111]. Besides the aforementioned effects, it has also been reported that bariatric surgery can also affect the levels of certain adipokines (such as leptin and adiponectin) and hepatokines (such as fetuin A), although the available evidence is limited and the results seem to vary depending the patient´s characteristics and the surgical intervention [97].

Studies have also demonstrated that bariatric surgery may trigger energy expenditure by increasing BAT sympathetic activity, and thus enhancing thermogenesis [112]. Other studies also reported that the potential damages that may occur in the vagal nerve during bariatric surgery result in changes on its hormonal sensitivity, altering the neuronal signalling.

Finally, it must be noted that surgical interventions usually involve the administration of antibiotics for prophylaxis and/or the management of potential post-surgery infectious complications [96]. Therefore, the above mentioned effects of bariatric surgery on gut microbiota should be interpreted with caution since these may vary depending on the usage of antibiotics.

### Other Approaches

**Fig. 3:**
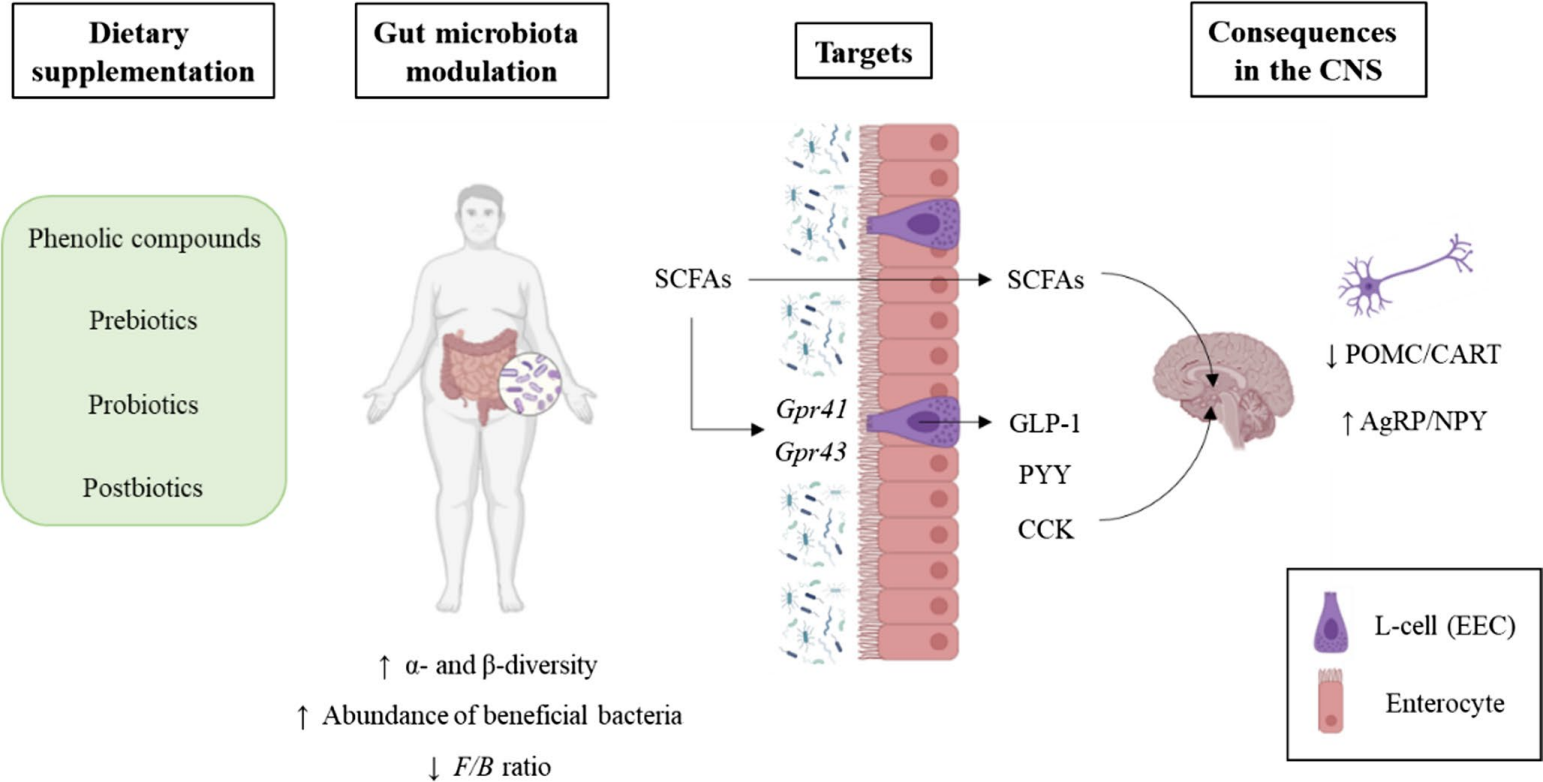
Schematic representation of the effects of phenolic compounds, prebiotics, probiotics and postbiotics on gut microbiota and consequently the gut-brain axis. AgRP: agouti-related protein, CART: cocaine- and amphetamine-regulated transcript, CCK: cholecystokinin, EEC: enteroendocrine cells, F/B: *Firmicutes/Bacteroidetes*, GLP-1: glucagon-like peptide-1, NPY: neuropeptide Y, POMC: pro-opiomelanocortin, PYY: peptide YY and SCFAs: short chain fatty acids

Although lifestyle modification and anti-obesity pharmacotherapy—together with bariatric surgery—remain the most effective and widely prescribed interventions for obesity management, alternative strategies have also been proposed. These include the administration of phenolic compounds as well as the use of viable or inactivated probiotics, both which have been shown to modulate gut microbiota composition (Fig. 3) [113–115]. Despite the accumulating evidence regarding the beneficial effects of the administration of phenolic compounds, prebiotics, probiotics, and postbiotics on gut microbiota composition, not in all these studies the effects of such interventions on the gut-brain axis have been addressed. Therefore, only studies providing data regarding the impact of these interventions on gut–brain axis have been included on this review).

#### Phenolic Compounds

Phenolic compounds are plant-derived second metabolites produced under normal and stress conditions [116]. These non-essential compounds are characterized by the presence of one or more aromatic rings and one or more hydroxyl groups and have been reported to have a potential anti-obesity effect [116]. Indeed, some studies have reported that phenolic compounds reduce food intake, thus driving weight and adiposity loss [117]. Although evidence from preclinical studies proposed the potential involvement of phenolic compounds in regulating the secretion of hormones able to signal along the gut-brain axis, thus affecting food intake and energy metabolism, the exact mechanisms of action are yet to be completely elucidated [117].

Preclinical studies conducted in rodents fed high-fat diets, shows that oral or intraperitoneal administration of phenolic compounds (either isolated or within extracts) reduce body-weight in most cases (Table 2) [118–124]. Interestingly, mixed results have been reported regarding the effect of these bioactive compounds on food intake, with some studies finding no changes [121, 122] and others reporting a significant reduction [118–120, 123, 124], even without an effect in body-weight [125]. Although not all studies have examined the effects of phenolic compounds on gut microbiota composition, those that did reported increases in the abundance of specific bacterial strains (*Bifidobacterium*, *Allobaculum*, *Parasutterella*, *Rombutsia* and *Akkermansia*, among others) in animals treated with pure compounds or mixed extracts [120, 121]. Moreover, Chang et al. [121] reported that the faecal content of SCFA was increased in animals fed a high-fat diet for 112 days and treated with 250 or 500 mg/kg of a gallic acid-rich extract. Of note, faecal SCFAs have been reported to play a significant role in the pathophysiology of obesity, by improving glucose tolerance, insulin sensitivity, gut barrier function and/or reducing inflammation [126]. Interestingly, some studies analysed the expression of key neuropeptides in the hypothalamus, finding an increased expression of *Pomc* and *Cart* transcripts (anorexigenic) and decreased mRNA levels or *Npy* and *Agrp* (orexigenic) in rodents treated with phenolic compounds and fed high-fat diets [118–121, 124, 125]. Of note, two studies reported that the administration of phenolic compounds modulated the secretion of intestinal hormones related to satiety signalling (GLP-1, CCK and PYY), as well as their mRNA expression in colonic tissues [120, 123]. These results suggest that the administration of phenolic compounds (isolated or in extracts) modulate gut microbiota composition and gut-hormone secretion, thus affecting the expression of hypothalamic regulators of appetite pathways.

**Table 2:** Selected preclinical studies (rodent models) addressing the effects of the administration of phenolic compounds, either isolated or within extracts, in obesity and the gut-brain axis

Reference	Animal model	Experimental conditions	Anti-obesity effects	Effects on the gut-brain axis
[118]	Male C57BL/6 mice	HFD (60% kcal from fat) + intraperitoneal injection of ferulic acid (10 mg kg^− 1^ BW for 12 weeks	↓ BW ↓ Adipocyte size and number	↓ Food intake ↓ mRNA levels of *AgRP* and *Npy* in hypothalamus
[119]	Male ICR mice	HFD (19.8% of fat) + peanut skin extract (4, 80 and 160 mg kg^− 1^ BW) for 6 weeks	↓ BW (80 and 160 mg/kg BW) ↓ eWAT weight (80 and 160 mg/kg BW) ↓ eWAT adipocyte size (80 mg/kg BW)	↓ Food intake (80 and 160 mg/kg BW) ↓ mRNA levels of *AgRP* and *Npy* (80 mg/kg BW) in hypothalamus
[124]	Male C57BL/6J mice	HFD (60% kcal from fat) + intraperitoneal injection of *Vitis vinifera* L. extract (400 mg kg^− 1^ BW) for 8 weeks	↓ BW ↓ WAT	↓ Food intake ↓ mRNA levels of *Npy* in serum and hypothalamus
[120]	Male C57BL/6 mice	HCD or HFHS (69% of kcal from carbohydrates) + gallic acid-rich apple polyphenol extract (125 or 500 mg kg^− 1^ BW) for 12 weeks	↓ BW ↓ scWAT adipocyte size	↓ Food intake ↑ mRNA levels of *Pomc* and *Cart* (HFHS + 125 or 500 mg/mg kg^− 1^ BW) in hypothalamus ↑ Plasma levels of GLP-1 and CCK ↑ mRNA levels of *Glp-1* and *Pyy* in colon
[123]	Male C57BL/6JRJ mice	HFD (60% kcal from fat) + polyphenol rich plant-extract (TOTUM-63) (2.7% w/w) for 10 weeks	↓ BW	↓ Food intake ↑ mRNA levels of *Pyy* in colon
[122]	Female Sprague Dawley rats	HFD (45% kcal from fat) + green tea polyphenols (0.5% w/vol in drinking water) for 4 months	↓ BW	No changes in food intake ↓ mRNA levels of *AgRP* in liver
[125]	Male Wistar rats	CD + Grape-seed proanthocyanidin extract (25 mg kg^− 1^ BW) for 21 days	No changes in BW	↓ Food intake ↑ mRNA levels of *Pomc* in hypothalamus
[121]	Male Sprague-Dawley rats	HFD (60% kcal from fat) + water extract of *Phyllanthus emblica* L. fruit (250 or 500 mg kg^− 1^ BW) for 112 days	↓ BW ↓ WAT weight	No changes in food intake ↑ mRNA levels of *Pomc* and *Cart* in hypothalamus ↓ mRNA levels of *AgRP* and *Npy* in hypothalamus

*AgRP *agouti-related peptide, *BW *body weight, *CCK *cholecystokinin, *CD *cafeteria diet, *eWAT *epididymal white adipose tissue, *HCD *high-carbohydrate diet, *HFD *high-fat diet, *HFHS *high-fructose high-sucrose diet, *kcal *kilocalories,*kg *kilogram, *mg *milligram, *Npy * neuropeptide Y, *Pomc *pro-opiomelanocortin, *Pyy *peptide YY, *rWAT *retroperitoneal white adipose tissue, *scWAT *subcutaneous white adipose tissue, *WAT *white adipose tissue, *WD *Western diet, *w/vol *weight to volume, *w/w*weight by weight, ↑ significant increase, ↓ significant decrease

As for clinical trials conducted in patients receiving phenolic compounds for obesity management, the available evidence remains inconclusive. While a recent systematic review reported that the supplementation with polyphenols reduced BMI, waist circumference (WC), visceral fat and total fat in individuals suffering from obesity [127], other authors observed changes in gut microbiota composition (increased abundance of *A. muciniphila* and *Bifidobacterium* spp.) and in bacterial function-related markers (increased SCFAs), but no changes in anthropometric variables [128]. These differences may be well due to variations in the type of intervention or to the presence of concurrent dietary and physical activity recommendations, which may increase the anti-obesity effect of the intervention. Although substantial evidence supports the anti-obesity potential of phenolic compounds, studies investigating their effects on gut–brain axis-related markers in individuals with obesity remain limited, albeit promising. In this regard, the administration of a caraway aqueous polyphenol extract for 90 days showed not only to reduce WC and waist to hip ratio (WHR) but also to diminish the appetite levels (measured using visual analogue scale method) of individuals with central obesity [129]. Additionally, an 8-week intervention study conducted with a combination of *Lippia citriodora* and *Hibiscus sabdariffa* showed that this polyphenol extract reduced BW, BMI and the percentage of body fat, with a concomitant amelioration of hunger and satiety feelings, and a higher concentration of plasmatic GLP-1 in individuals with obesity [130]. Interestingly, another clinical trial conducted with the same plant extract (*Lippia citriodora* and *Hibiscus sabdariffa*) for 60 days, reported a significant decrease in fat mass and lower levels of leptin, as well as a greater satiety and lower *ad libitum* food intake in participants receiving the phenolic compound-rich capsules compared to those in the placebo group [131]. Although the results regarding leptin might seem contradictory, as it is an anorexigenic hormone and the authors observed a reduced food intake, the lower fat mass could explain these results, considering that leptin is mainly synthesized in WAT. As GLP-1 levels were higher in the treated group compared to baseline, authors suggested that the treatment might modulate GLP-1 secretion, modulating food intake and thus influencing body-weight in this case [131]. Despite the treatments employed in the aforementioned clinical trials consisted of polyphenol extracts, representing a limitation for elucidating the specific mechanisms of action of individual phenolic compounds, they more closely resemble the manner in which these bioactive compounds are typically consumed within a habitual dietary pattern.

#### Prebiotics, Probiotics, Synbiotics and Postbiotics

Another approach to addressing the chronic metabolic disturbances associated with obesity-induced gut dysbiosis involves treatments that directly modulate gut microbiota composition or the profile of microbiota-derived metabolites, which in turn have been shown to influence feeding behaviour via the gut–brain axis [132]. The administration of prebiotics, probiotics, synbiotics or postbiotics has therefore emerged as a promising tool for obesity management (Table 3).

**Table 3:** Selected preclinical studies (rodent models) addressing the effects of the administration of prebiotics, probiotics, synbiotics or postbiotics in obesity and the gut-brain axis

	Reference	Animal model	Experimental conditions	Anti-obesity effects	Effects on the gut-brain axis
Prebiotic interventions	[133]	Male ICR mice	HFD (45% kcal from fat) + 10% fructose in drinking water + 100 or 200 mg/kg/day mannose oligosaccharide for 4 weeks	↓ BW gain	↓ Food intake ↑ mRNA levels of *Glp-1* and *Pyy* in colon ↓ mRNA levels of *Npy* and *AgRP* in hypothalamus ↑ mRNA levels of *Pomc* and *Cart* in hypothalamus
	[134]	Male C57BL/6J mice	HFD (60% kcal from fat) + 10% oligofructose in drinking water for 6 weeks	↓ BW ↓ Fat mass ↓ scWAT and eWAT weight	NS food intake ↑ mRNA levels of *proglucagon* in cecum and colon
	[135]	Male C57BL/6J mice	HFD (60% kcal from fat) + 10% 2’-fucosyllactose in drinking water for 6 weeks	↓ BW ↓ Fat mass ↓ scWAT. eWAT and VAT weight	NS food intake ↑ Serum levels of GLP-1 and PYY
	[136]	Male Sprague Dawley rats	HFSD + 10% (wt/wt) oligofructose for 8 weeks	↓ BW ↓ % Fat mass ↓ % Body fat	↓ Food intake ↑ Serum levels of GLP-1
Probiotic interventions	[137]	Male C57BL/6J mice	HFD (60% kcal from fat) + VSL#3 (5 mg/kg bw) for 8 weeks	↓ BW ↓ Fat mass ↓ eWAT weight	↓ Food intake ↑ Serum levels of GLP-1
	[138]	Male C57BL/6J mice	HFD (60% kcal from fat) + *L. salivarius* LCK 11 (1 × 10^7^ CFU/day)	↓ BW ↓ iWAT, pWAT and eWAT weight	↓ Food intake ↑ mRNA levels of *Pyy* in ileum, colon and rectum ↑ protein levels or PYY in rectum ↑ Serum levels of PYY
	[139]	Male C57BL/6J mice	HFD (60% kcal from fat) + *F. prausnitzii* A2-165, EB-FPDK3, EB-FPDK6, EB-FPDK11 or EB-FPYYK1 (1 × 10^8^ CFU/day) for 12 weeks	↓ BW ↓ AT weight ↓ scWAT, mWAT and eWAT	↓ Food intake ↑ mRNA levels of *Pyy* (all), *Glp-1*,* Gpr43 (*A2-165 and DK11*)* and *Gpr41 (*A2-165 and DK9*)* in colon ↑ mRNA levels of *Ckk*,* Pyy* (A2-165, DK3 and DK11) in jejunum ↓ mRNA levels of *Npy* (all) and *AgRP* (A2-165 and YK1) in hypothalamus ↑ mRNA levels of *Cart* (A2-165, DK3, DK9 and DK11) and *Pomc* (DK3 and DK11) in hypothalamus
	[140]	Male C57BL/6J mice	HFD (60% kcal from fat) + *L.rhamnosus* HF01 (10^9^ CFU/100 g/bw) for 12 weeks	↓ BW ↓ sWAT fat index	↓ Food intake ↑ mRNA levels of *Pyy*,* Grp41* and *Grp43* in colon ↑ Serum levels of PYY
Synbiotic interventions	[141]	Male Wistar rats	HFD (60% kcal from fat) + *L. paracasei* HII01 (10^8^ CFU/day) + 10% xylooligosaccharide in PBS for 12 weeks	↓ BW ↓ VAT weight	NS food intake ↓ MDA levels in brain ↓ ROS levels in brain and hippocampus ↓ mRNA levels of *Bax* in hippocampus ↑ mRNA levels of *Bcl-2* in hippocampus
	[142]	Male albino rats	HFD (43% kcal from fat) + *L. acidophilus* (1 × 10^10^ CFU/day) + 2 & inulin fructooligosaccharides for 6 weeks	↓ BW	NS food intake ↑ Serum levels of GLP-1

*AgRP* agouti-related peptide, *Bax* BCL2 associated X, *BW* body weight, *Cart *cocaine and amphetamine regulated transcript,*CFU* colony-forming unit, *eWAT* epididymal white adipose tissue, *GLP-1* glucagon-like peptide- 1, g gram, *Grp41/43* free fatty acid receptors, *HFD* high-fat diet, HFSD high-fat sucrose diet, *iWAT*inguinal white adipose tissue, *kcal* kilocalories, *kg* kilogram, *MAD* malondialdehyde, *mWAT* mesenteric white adipose tissue, *Npy* neuropeptide Y, *NS* non-significant, *Pomc* pro-opiomelanocortin,* pWAT* perirenal white adipose tissue, *PYY* peptide YY, *ROS* reactive oxygen species, *scWAT* subcutaneous white adipose tissue,* VAT*visceral white adipose tissue, *wt/wt *weight by weight, ↑ significant increase, ↓: significant decrease

One of the approaches targeting gut microbiota for obesity management is the administration of prebiotics. Prebiotics have been defined as non-digestible food ingredients that stimulate the growth and/or activity of specific colonic bacteria, thus improving host health [143]. The administration of prebiotics prevents body-weight gain through different mechanisms of action [144]. Interestingly, although some authors have reported that prebiotic administration reduces food intake, which partly explains the lower diet-derived body-weight gain [133, 136], others reported no changes in energy intake in rodents fed obesogenic diets [134, 135]. Regardless of observing changes in food intake, most of the available studies conducted in diet-induced obese rodent models have reported changes in gut microbiota composition and function. More specifically, in a study testing mannose oligosaccharide administration for obesity treatment on mice fed a western diet (45% of fat and 10% fructose-upplemented watr), an increase in the relative abundance of butyrate-producing bacteria (including *Roseburia*, *Faecalibaculum* or *Butyricicoccus*) was observed [133]. Indeed, higher serum butyrate levels, accompanied by lower levels of transcripts of orexigenic hormones (*Npy* and *AgRP*), and by increased mRNA levels of anorexigenic neuron transcripts (*Pomc* and *Cart*) and leptin receptor were reported [133]. Oligofructose supplementation has also been shown to reduce body-weight and adiposity in diet-induced obese rats. In this case, a higher abundance of *Bacteroides*, *Lactobacillus* and *Bifidobacterium* species, along with a decrease on the presence of bacterial members from the *Clostridium* and *Enterobacteriaceae* species were observed [136]. Additionally, an increase in the abundance of *Bacteroides* and *Verrucomicrobia* species, among others, was observed in studies supplementing mice with prebiotics (oligofructose or 2’-fucosyllactose) [134, 135]. These changes were accompanied by higher GLP-1 and PYY serum levels, as well as higher mRNA expression levels of proglucagon, a GLP-1 precursor, in cecum and colon samples [134–136].

In clinical trials, prebiotic administration has been reported to not reduce body weight or adiposity in individuals with overweight or obesity [145]. Interestingly, despite the lack of effects on body weight, Rahat-Rozenbloom et al. [146] stated that individuals with obesity displayed a different EEC structure and thus hormone secretion. In this line, other studies have reported that, although individuals with excessive body fat display more EECs, the release of hormones signalling satiety is lower compared to lean subjects [147]. The authors suggested that prebiotic administration may lead to changes in gut structure and microbiota composition, thus affecting hormone secretion as observed in preclinical models [146]. Moreover, acute prebiotic supplementation has been shown to mildly influence appetite related signals (higher levels of SCFAs and lower ghrelin response) [146, 148]. However, further research is needed in order to describe how prebiotics modulate gut microbiota composition, and how these changes affect the secretion of mediators involved in the regulation of gut-brain axis.

An additional strategy for obesity management is the administration of probiotics. Probiotics are defined as live microorganisms that, when administered in adequate amounts, may exert health benefits in the host [149]. Probiotics have traditionally been used to improve digestive function, manage food allergies, and treat gastrointestinal disorders. In preclinical models, probiotic interventions have also shown efficacy in obesity management—improving glycaemic control and lipid metabolism, attenuating inflammation, and modulating gut microbiota composition [150]. Interestingly, some authors have reported that administering different probiotic strains in varying doses (from 1 × 10^8^ to 1 × 10^9^ CFU/day) to mice fed unbalanced diets (60% energy oming from fat) during different experimental periods (from 8 to 12 weeks), significantly reduces body-weight gain concomitantly with a decrease in food intake [137–140]. As one of the mechanisms of action underlying the beneficial effects of probiotic administration in host metabolism is their ability to modulate gut microbiota composition, some authors have explored the compositional and functional changes that gut microbiota undergoes after viable bacteria supplementation. Preclinical studies reporting a lower food intake in animals supplemented with probiotics, observed a reduction in *Firmicutes/Bacteroidetes* ratio [138, 140], or changes in the relative abundance of specific bacteria (increased abundance of *Ruminococcus*, *rc4-4*, *Parabacteroides*, *Lactococcus* and *Bacteroides*) [139]. These changes in gut microbiota composition were accompanied by differences in microbial functionality. In other study, Sun et al. [140]. reported that mice supplemented with *L. rhamnosus* HF01 and fed a high-fat diet displayed a more functional gut microbiota community, with higher efficiency for protein and carbohydrate digestion and absorption. Apart from their effect on gut microbiota composition, probiotics have also been shown to modify the levels of microbiota-derived SCFAs. Indeed, higher levels of SCFAs (propionic, butyric, isobutyric and isovaleric), and of the expression of their receptors (*Gpr41* and *Gpr43*) in colon have been described in animals treated with probiotics [140].

Beyond their local gastrointestinal effects, probiotics may also confer benefits by modulating the gut–brain axis. Studies have shown that probiotics enhance the gene expression of satiety hormones (such as *Pyy*, *Glp-1* and *Cck*) in the colon, and to increase the gene expression of anorexigenic neurons (*Pomc* and *Cart*), while reducing the mRNA expression of orexigenic neurons (*Npy*) in the hypothalamus [137–140]. Additionally, other authors have reported changes in the serum levels of hormones related to satiety. More specifically, the administration of viable bacteria has been shown to increase serum GLP-1 and PYY levels in mice fed a high-fat diet and supplemented with probiotics [137, 138]. In light of these results, it could be hypothesized that probiotic administration prevents high-fat diet-induced obesity partly by influencing the secretion of hormones related to satiety regulation in various tissues, and by regulating the expression of certain neuronal pathways. The link between probiotic administration, changes in SCFA production and the modulation of appetite signalling has been confirmed in some studies, such as that conducted by Yadav et al. [137]., who observed higher levels of faecal butyrate in mice treated with VSL#3 probiotic preparation. These authors reported that such change was related to an increased abundance of butyrate-producing bacteria. A further analysis of the interaction of butyrate and the enhanced levels of satiety hormones (GLP-1), demonstrated that butyrate promotes the expression of genes related to GLP-1 synthesis and secretion on EEC-human cultures [137]. Additionally, higher *Gpr41* mRNA levels were measured in this cell line, suggesting a feasible role of butyrate on promoting its expression, and thus, influencing EEC hormone secretion [137].

Available clinical trials assessing the effects of probiotics in body-weight reduction and markers related to gut-brain axis and appetite regulation are scarce. Contrary to that observed in preclinical studies, clinical trials conducted in individuals with overweight and obesity supplemented with probiotics did not report changes in energy intake nor in body-weight [151, 152]. However, Schellekens et al. [151]observed differences between the subpopulations of study participants, finding that the probiotic intervention (*B. longum*, 1 × 10^10^ CFU/day for 12 weeks) reduced the cortisol awakening response in individuals with obesity, but not in individuals with overweight. Of note, cortisol-awakening response may influence appetite and food choices, being that higher values are associated with an increased appetite and calorie intake [153]. Moreover, active ghrelin levels, which are normally dysregulated in obesity, were normalized in individuals with obesity supplemented with the probiotic [151]. Considering the data obtained in preclinical studies regarding the effects of probiotic administration in markers related to satiety pathways, it could be hypothesized that the anti-obesity effect of viable bacteria might be mediated through the modulation of gut-brain axis pathways. However, more research is needed to clarify the underlying mechanisms in humans.

Considering the reported benefits of prebiotic and probiotic administration for obesity management, it is worth noting that their combined supplementation in the form of synbiotics has also yielded promising results. Synbiotics are defined as a mixture of live microorganisms (probiotics) and substrate(s) selectively utilized by host microorganisms (prebiotics) that confers a health benefit on the host [154]. The rational behind synbiotic use relies on the potential of prebiotics to enhance probiotic survival throughout the intestinal tract, while also exerting beneficial effects on the host gut microbiota community [155].

Administration of synbiotics has shown beneficial effects in both the prevention and treatment of obesity in diet-induced preclinical rodent models. Specifically, supplementation of animals fed obesogenic diets (40–60% of energy from fat) with single or mixed bacteria strains at different doses (5 × 10^7^−10^9^ CFU/day), combined with prebiotics and administered over variable experimental periods (6 to 12 weeks), significantly reduces body weight gain, total body weight adipose tissue weight or fat mass percentage, without affecting food intake [141, 142, 156–163]. Similarly to studies administering prebiotics or probiotics alone, synbiotic supplementation has been shown to modulate gut microbiota composition, which may represent an key mechanism underlying its beneficial effects on obesity. In this context, several studies have reported that synbiotics increase gut microbiota diversity (as assessed by the Shannon index) [157, 158], decrease *Firmicutes*/*Bacteroidetes* ratio [160, 163], and alter the relative abundance of specific bacterial taxa (including reductions in *Proteobacteria* and *Enterobacteria*, and increases in *Actinobacteria*) [158, 160, 163]. These microbiota changes were accompanied by a significant increase in the cecal concentrations of total SCFAs [142], or in propionate and/or acetate following synbiotic administration [156, 157]. Despite the strong evidence supporting the beneficial effects of synbiotic administration in attenuating the deleterious metabolic consequences of obesogenic diets in preclinical rodent models, information regarding the effects of these formulations on markers related to gut-brain axis regulation remains limited. In this regard, Kafoury et al. [142]. reported that 6 weeks of synbiotic treatment in diet-induced obese rats, attenuated high-fat diet-derived impairments in anthropometric parameters (decreased final body weight) and increased circulating levels of GLP-1 and serotonin. In addition, Chunchai et al. [141]. demonstrated that a combination of *L. paracasei* (1 × 10^8^ CFU) and xylooligosaccharide (10% in PBS) for 12 weeks significantly reduced high-fat diet-induced brai oxidative damage and exerted anti-apoptotic effects in the hippocampus of rats. Given the limited available evidence, further studies are warranted to fully elucidate the potential mechanisms through which synbiotics may modulate gut-brain axis pathways in the context of obesity in preclinical models.

With respect to clinical evidence, studies conducted in adults with obesity and supplemented with synbiotics for varying durations (8 to 12 weeks), have reported heterogeneous outcomes. While some trials demonstrated reductions in anthropometric parameters (including body weight, body fat mass or waist circumference) following synbiotic supplementation [164, 165], others reported no significant changes in these measures [166, 167]. Notably, in studies reporting no anthropometric improvements, synbiotic supplementation still induced significant alterations in gut microbiota composition, including increased microbial richness and higher relative abundance of beneficial taxa such as *Bifidobacterium* and *Lactobacillus* [166, 167]. Beyond microbiota modulation, Chaiyasut et al. [165]. reported increased fecal concentrations of SCFAs following 12 weeks of supplementation with a synbiotic formulation combining two bacteria strains, fructooligosaccharides, and inulin. Regarding gut-brain axis-related markers, Rabiei et al. [164]. observed a significant increase in circulating GLP-1 levels re in adults with obesity receiving synbiotic supplementation in combination with energy restriction. Furthermore, synbiotic administration was shown to attenuate obesity-associated neuroinflammation, as indicated by a reduced urinary quinolinic acid/5-hydroxyindoleacetic acid (QA/5-HIAA) ratio after 12 weeks of treatment [165].

Although probiotics have been proven effective for the management of obesity, the administration of viable bacteria may pose risks, such as systemic infections, deleterious metabolic activities, excessive immune stimulation or gene transfer in vulnerable individuals [168]. More recently the usage of postbiotics, defined as a preparation of inanimate microorganisms and/or their components that confers a health benefit on the host [169], has gained special attention. Postbiotics include inactivated bacteria, SCFAs, microbial fractions, bacteria-derived proteins or enzymes, secreted or extracellular polysaccharides and lipoteichoic acids, among others [170]. Compared to probiotics, postbiotics present several advantages in terms of security, as well as improved techno-functional characteristics (easier storage, transport and manufacturing) [171]. As for their metabolic effects, in a recent review article, inactivated bacteria have been reported to exert benefits in both preclinical obese models and clinical trials including individuals with overweight/obesity [115]. Interestingly, the anti-obesity effects of non-viable bacteria were shown to be similar to those exerted by their viable counterparts [115].

Considering that some of the approaches targeting gut microbiota composition for obesity management promote gut hormone secretion by enhancing SCFA production, some authors have tested the administration of SCFAs as a postbiotic-based therapy to prevent obesity. Concerning this issue, SCFA-administration has been reported to reduce diet-induced body-weight gain in preclinical rodent models [172–174]. As for the mechanisms of action, the administration of single SCFAs (sodium butyrate at 5% w/w) o mixtures (indole 3-propionic acid, sodium butyrate and valeric acid) for experimental periods ranging from 14 days to 9 weeks, has been reported to reduce food intake in rodents fed high-fat diets (45 to 60% of enegy as fat) [172–174]. While not all studies analysed the effects of SCFA administration on gut microbiota composition, some authors have observed that the administration of sodium butyrate or a SCFA mixture (indole 3-propionic acid, sodium butyrate and valeric acid) increased the abundance of bacteria from the phylum *Firmicutes* and/or the α- and β-diversity [172, 173]. The link between lower food intake, changes in gut microbiota composition and expression of certain markers related to gut-brain axis satiety pathways has also briefly been analysed. Li et al. [172] reported that a single exposure to sodium butyrate by oral administration was sufficient to reduce food intake, as well as hypothalamic neuronal signalling (lower neuronal activation and lower NPY-positive neurons) in mice fed a high-fat diet. The beneficial effects of butyrate were further explored, showing that chronic administration of sodium butyrate for 9 weeks increased the sympathetic outflow towards BAT (thus increasing thermogenesis) [172]. Additionally, Dong et al. [173] reported that treating animals with a SCFA mixture (indole-3 propionic acid, sodium butyrate and valeric acid) decreased both, the levels of peripheral ghrelin and the expression of *AgRP*, *Npy* and *Orexin* mRNA transcripts in the hypothalamus (appetite stimulators). It also increased the circulating levels of insulin and PYY as well as the mRNA expression of *Pomc* in the (appetite inhibitors) [173].

The available studies have analysed the effects of SCFAs when administered in capsules, by colonic infusions (enema) or combined with food products in individuals with overweight or obesity [175–178]. While some studies have analysed the effects of an acute administration of SCFAs [176, 178], others have registered the outcome of chronic exposure (from 7 to 60 days) [177, 178]. Interestingly, although the administration of inulin-propionate ester for 7 days increased BW, fat free mass and total content of body water were also higher in the group receiving the treatment [177]. Despite a tendency to a lower energy intake was observed in participants supplemented with inulin-propionate, no changes in serum levels of GLP-1 or PYY were reported [177]. By contrast, in the study of Amiri et al. [175], 60 days of sodium butyrate supplementation combined with a hypocaloric diet reduced BW, WC and BMI to the same extent as placebo. Moreover, no changes were observed neither in GLP-1 levels nor in appetite sensation-related markers between the treated and placebo groups [175]. These results suggest that sodium butyrate did not exert additional beneficial effects on weight loss when combined with a calorie restrictive diet. However, given the scarcity of studies analysing the direct effects of SCFAs on appetite regulating hormone levels, more research is needed to clarify the underlying mechanisms of action, as well as the feasible future practicality of these kind of treatments for obesity management. There are also studies that have analysed the effectiveness of postbiotics other than SCFAs, such as muramyl dipeptide, urolithin A and B, probiotic derived extracellular vesicles, surface layer proteins, bacterial exopolysaccharides or bacteroicins, in obesity management [179]. Interestingly, the majority of these studies (conducted in animal models of obesity) have demonstrated the effectiveness of the administration of these postbiotics in the management (mainly prevention) of obesity. Moreover, these studies have also addressed the mechanisms of action underlying the effects exerted by postbiotic administration, such as thermogenesis induction and browning of WAT, reduction of pro-inflammatory cytokine levels or the activation of the AMPK signalling pathway, among others [179]. However, to the best of our knowledge, in none of these studies the effect of postbiotic administration in the gut-brain axis has been analysed, and thus, have not been included in this review article.

An additional treatment considered as postbiotic that has been reported to exert beneficial effects in preventing obesity, is the administration of inactivated bacteria. Although the evidence is scarce, Ashrafian et al. [180] reported that administering pasteurized *Akkermansia muciniphila* (10^9^ CFU/200 µL) to mice fed a high-fat diet (60% caories from fat) for 5 weeks significantly reduced body-weight and food intake [180]. Interestingly, this study compared the effects of live and pasteurized bacteria administration, finding that the effect of the postbiotic lowering body-weight was greater compared to the probiotic [180]. Although there are other studies conducted with pasteurized *A. muciniphila* for longer experimental periods (12 weeks) that have also observed a reduction in body-weight after non-viable bacteria administration, the authors did not report information about food intake [181]. Postbiotics from *A. municiphila* (pasteurized bacteria and its extracellular vesicles) have shown to modify gut microbiota composition, by lowering the abundance of *Firmicutes* and *Firmicutes*/*Bacteroidetes* ratio [180]. Regarding the effects of non-viable bacteria administration in the expression of markers related to gut-brain axis regulation, Choi et al. [181] reported no changes in the intestinal mRNA expression levels of *Pyy* and *Glp-1*. Given the limited understanding of the mechanisms underlying the appetite-suppressing effects of postbiotics in obese rodent models, further research in this area is warranted.

To our knowledge, no clinical trials have been published addressing the effects of non-viable bacteria on appetite-related markers. However, a study analysing the effects of a postbiotic-supplemented drink (200 mg of heat-killed *L. amylovorus* CP1563) in individuals with overweight showed that after 12 weeks, body-weight and fat mass were reduced [182]. Interestingly, these changes were observed along with modifications in gut microbiota composition, with an increase in the relative abundances of *Roseburia* and in an unknown genus from *Lachnospiraceae* family (namely *Lachnospiraceae*; *g*) [182]. As some members of *Lachnospiraceae* and *Roseburia* families have been characterised as butyrate-producing bacteria, authors suggested that the beneficial effects of the treatment could be mediated by an increase in butyrate levels, as this SCFA has been reported to regulate appetite within the gut-brain axis [182].

Overall, the available evidence indicates that the usage of interventions directly tackling the gut microbiota (modulating its composition and/or the profile of bacterial metabolites) is an effective approach for obesity management. Nevertheless, there are still knowledge gaps in this field for the development of more personalized and/or efficient therapeutic tools. For instance, the majority of studies investigating the effectiveness of probiotic administration for obesity management did not analyse the effect of this intervention in the gut-brain axis. Therefore, it is still not possible to stablish whether the administration of combined strains could be a more effective approach than the administration of a single bacterial strain. In the case of the administration of inactivated bacteria for obesity management, despite this approach has demonstrated to be at least as effective as the administration of their viable probiotic counterparts (being in some cases even more effective), it is not possible to elucidate which could be the bacterial components responsible of the anti-obesity effects. This, in turn, represents a limitation in order to develop efficient and personalized treatments based in the administration of the most effective components. In order to overcome this limitation, several studies have been carried out using specific bacterial components and analysing their effects in obesity (mainly in preclinical studies). However, to the best of our knowledge only studies using SCFAs have investigated the effects of this approach in the gut-brain axis, and thus, the current knowledge is still limited. In addition, all studies conducted with postbiotics (regardless whether they analysed the gut-brain axis) have only used a single bacterial component or inactivated bacterial strain as intervention, and thus, it is not possible to know if the administration of combined postbiotics (different bacterial components, different inactivated bacterial strains or the combination of bacterial components and inactivated bacteria) could exert additional or synergistic effects. This issue is especially relevant since, according to the available literature, the mechanisms of action underlying the anti-obesity effects of different postbiotics differ, and thus it cannot ruled out that the combination of postbiotics may result in the up/down-regulation of complementary metabolic pathways.

## Conclusions

Preclinical and clinical evidence increasingly supports a central role of the gut–brain axis in the onset and progression of obesity, influencing both energy intake and utilization through diverse mechanisms. Studies examining alterations in gut–brain communication during obesity treatment further underscore the relevance of this signalling network for disease management. Nonetheless, data on the therapeutic implications of targeting the gut–brain axis remain limited, particularly from clinical trials. Future research is therefore essential to clarify the mechanistic underpinnings and translational relevance of these pathways, ultimately enabling the development of more effective and personalized therapeutic strategies for obesity.

## Data Availability

No datasets were generated or analysed during the current study.

## Key References

- Peluzio M do CG, Martinez JA, Milagro FI. Postbiotics: Metabolites and mechanisms involved in microbiota-host interactions. Trends Food Sci Technol. 2021;108:11–26. doi:10.1016/j.tifs.2020.12.004
  - ○ This article comprehensively analyses the described health implications of an array of postbiotics, detailing the mechanisms of action and potential benefits on human health. Therefore, provides a great body of evidence on this topic.
- Longo S, Rizza S, Federici M. Microbiota-gut-brain axis: relationships among the vagus nerve, gut microbiota, obesity, and diabetes. Acta Diabetol. 2023;60:1007–17. doi: 10.1007/s00592-023-02088-x
  - ○ This article is an important review that comprehensively addresses the involvement of the microbiota-gut-brain axis in the development of obesity and type 2 diabetes.
- Chakhtoura M, Haber R, Ghezzawi M, Rhayem C, Tcheroyan R, Mantzoros CS. Pharmacotherapy of obesity: an update on the available medications and drugs under investigation. EClinicalMedicine. 2023;58: 101882. doi: 10.1016/j.eclinm.2023.101882
  - ○ In this article, an extensive revision of the available drugs for obesity pharmacotherapy is carried out. Of note, besides explaining the mechanisms of action of these drugs, this article also comprehensively explains their implication on body weight and adipose tissue weight reductions. Consequently, this review article provides a great an updated body of evidence on this topic.
- Amiri P, Hosseini SA, Saghafi-Asl M, Roshanravan N, Tootoonchian M. Expression of PGC-1α, PPAR-α and UCP1 genes, metabolic and anthropometric factors in response to sodium butyrate supplementation in patients with obesity: a triple-blind, randomized placebo-controlled clinical trial. Eur J Clin Nutr. 2025;79:249–57. doi:10.1038/s41430-024-01512-x
  - ○ This interesting article investigates the effects of sodium butyrate supplementation in anthropometric and metabolic risk variables associated with obesity in obese subjects in a hypocaloric diet. Interestingly, the mechanism of action underlying the potential effects of this intervention were also analysed.
